# Supplementary figures and images for: Genomic Complexity and Complex Chromosomal Rearrangements in Genetic Diagnosis: Two Illustrative Cases on Chromosome 7
Source: Genes (Basel). 2023 Aug 27;14(9):1700. doi: 10.3390/genes14091700 (PMC10530880; doi:10.3390/genes14091700)

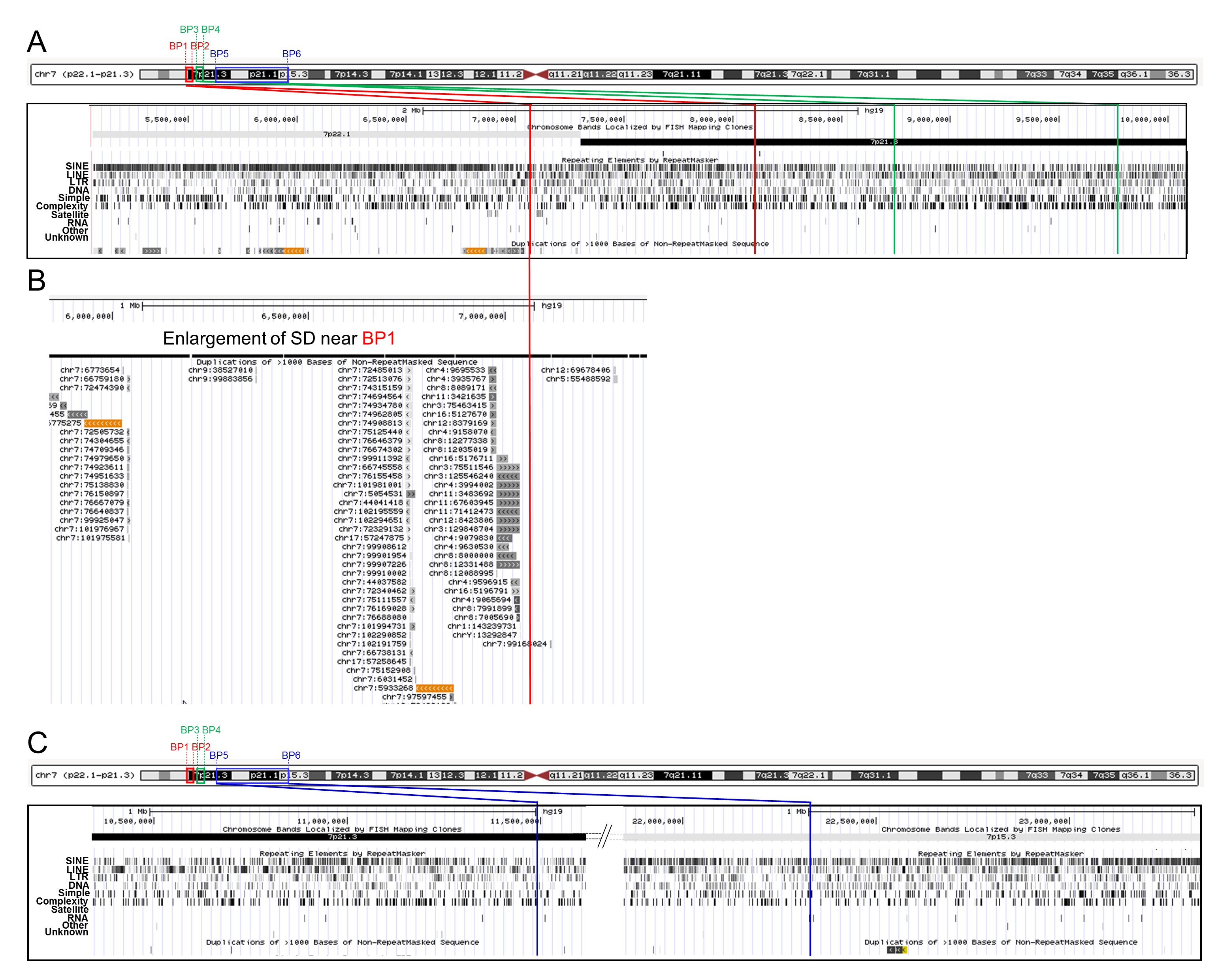

Supplement: Supplementary file 1 [file genes-14-01700-s001.zip › FigS1.jpg]

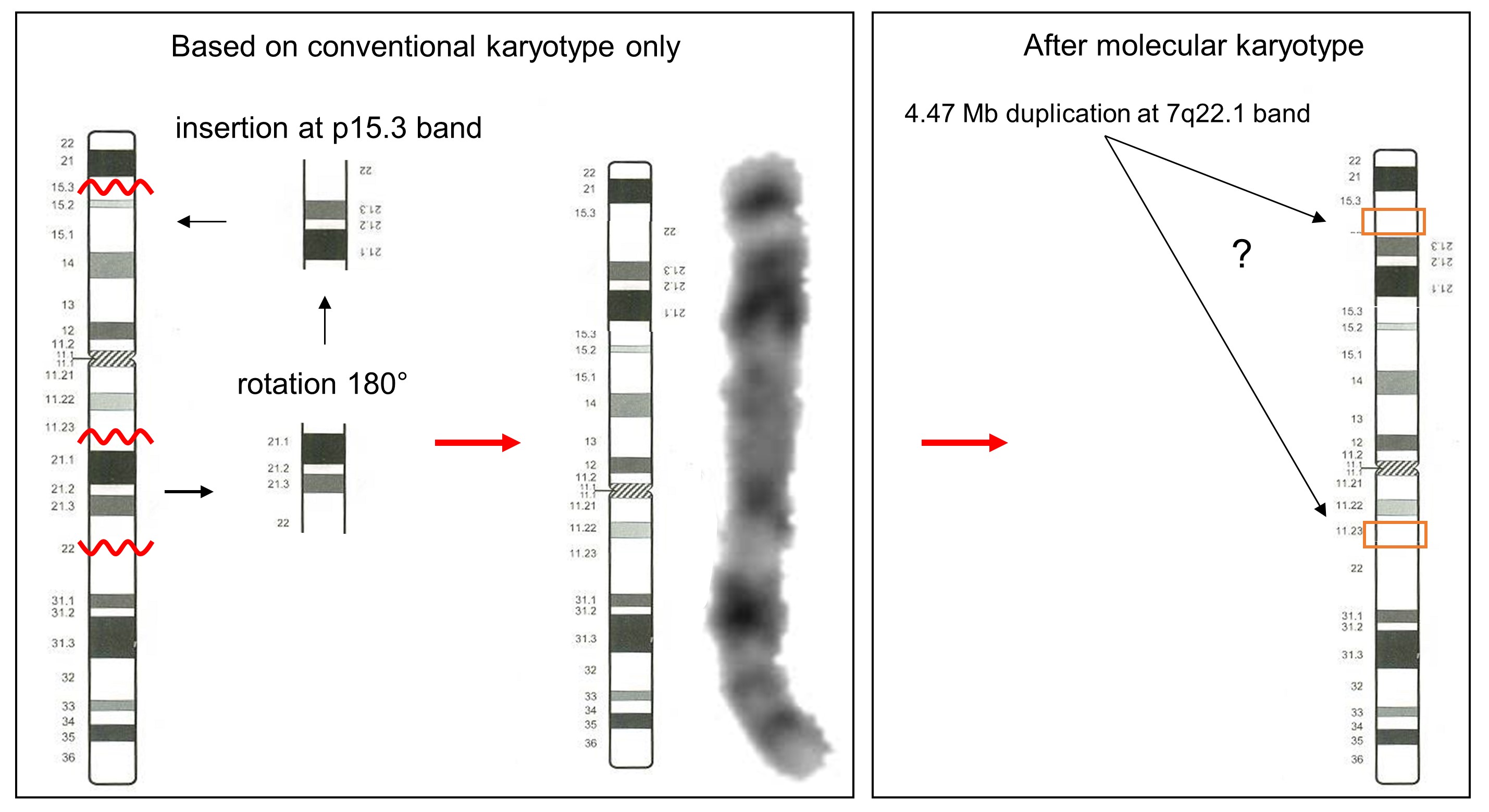

Supplement: Supplementary file 1 [file genes-14-01700-s001.zip › FigS2.jpg]

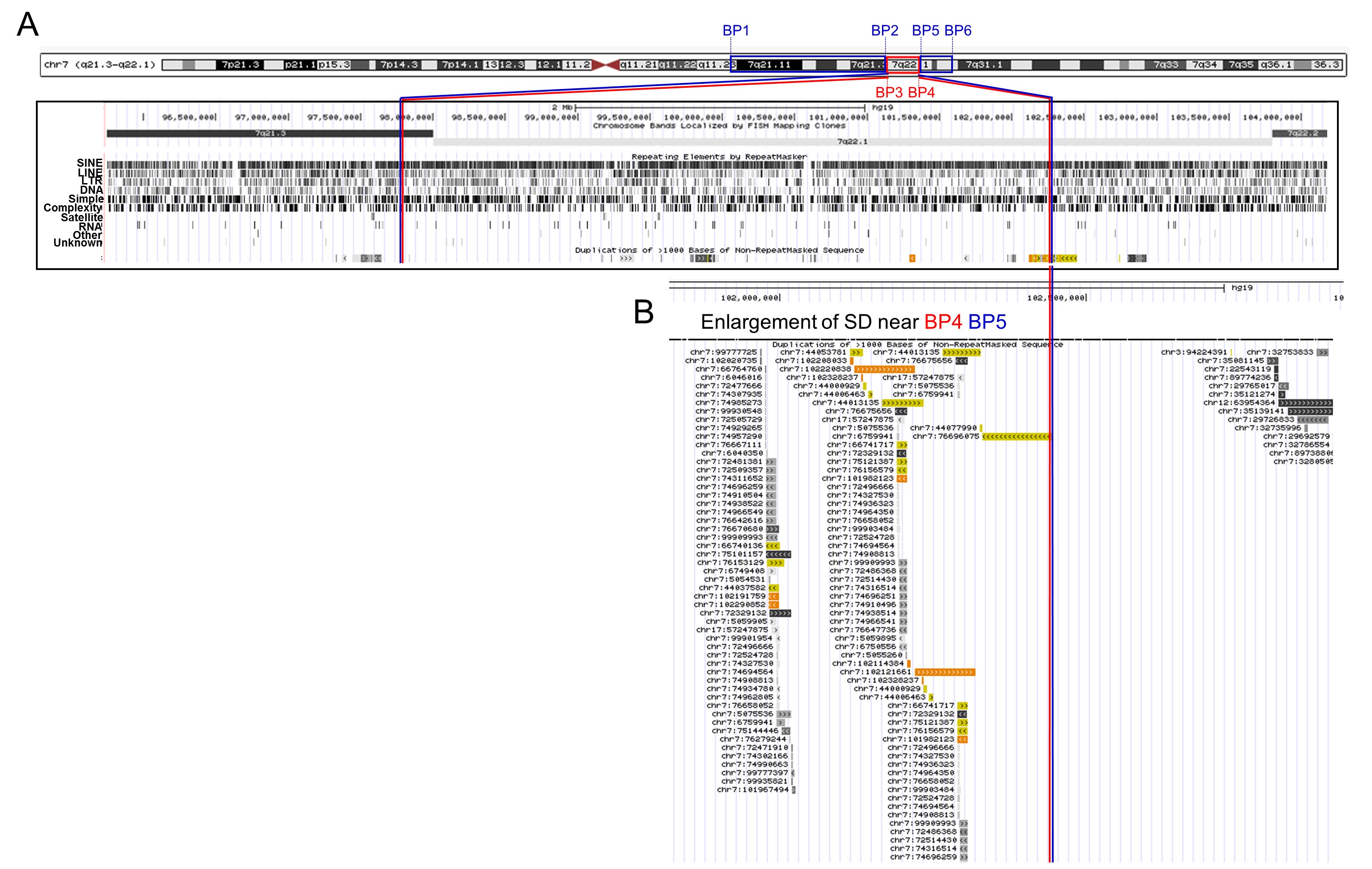

Supplement: Supplementary file 1 [file genes-14-01700-s001.zip › FigS3.jpg]

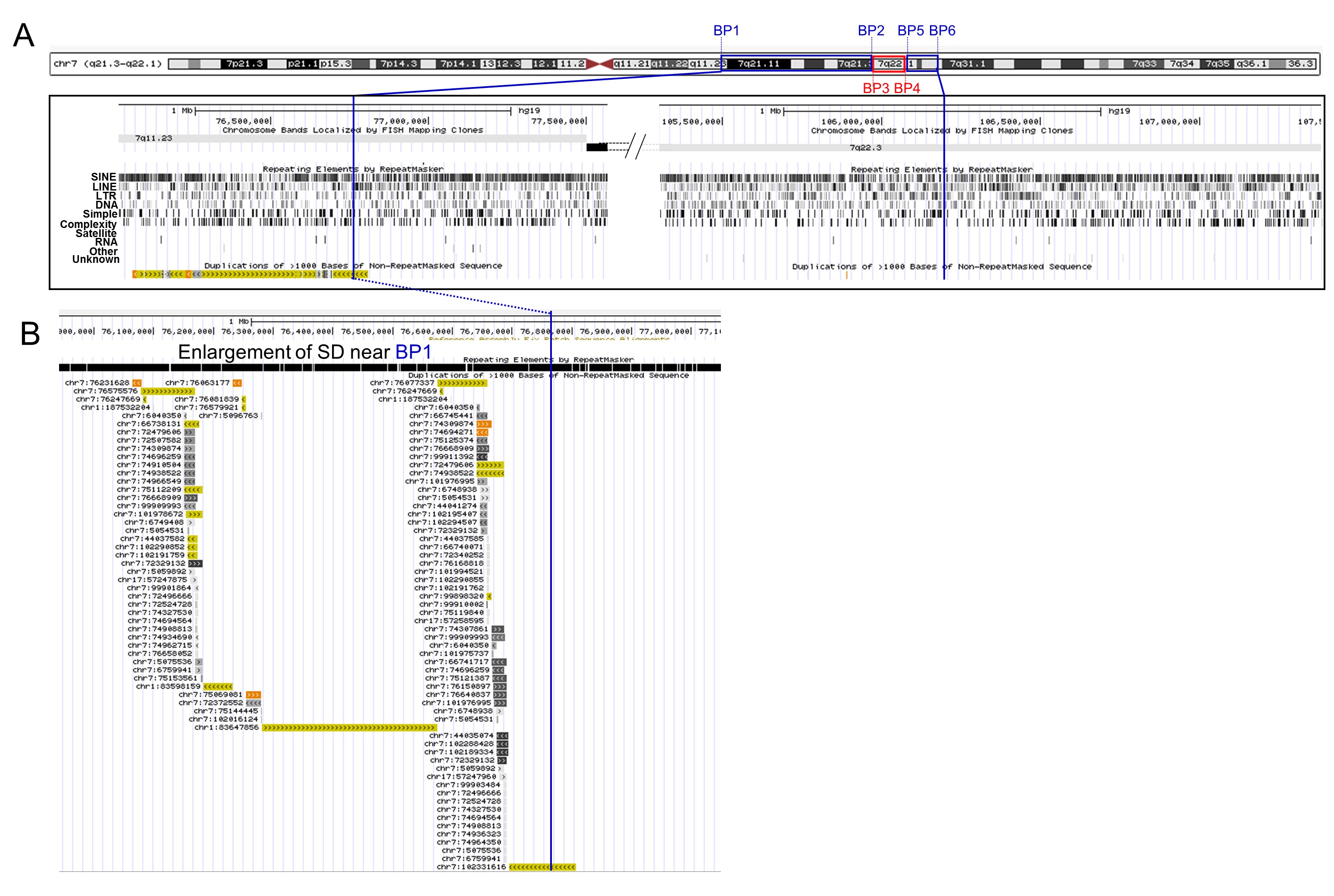

Supplement: Supplementary file 1 [file genes-14-01700-s001.zip › FigS4.jpg]

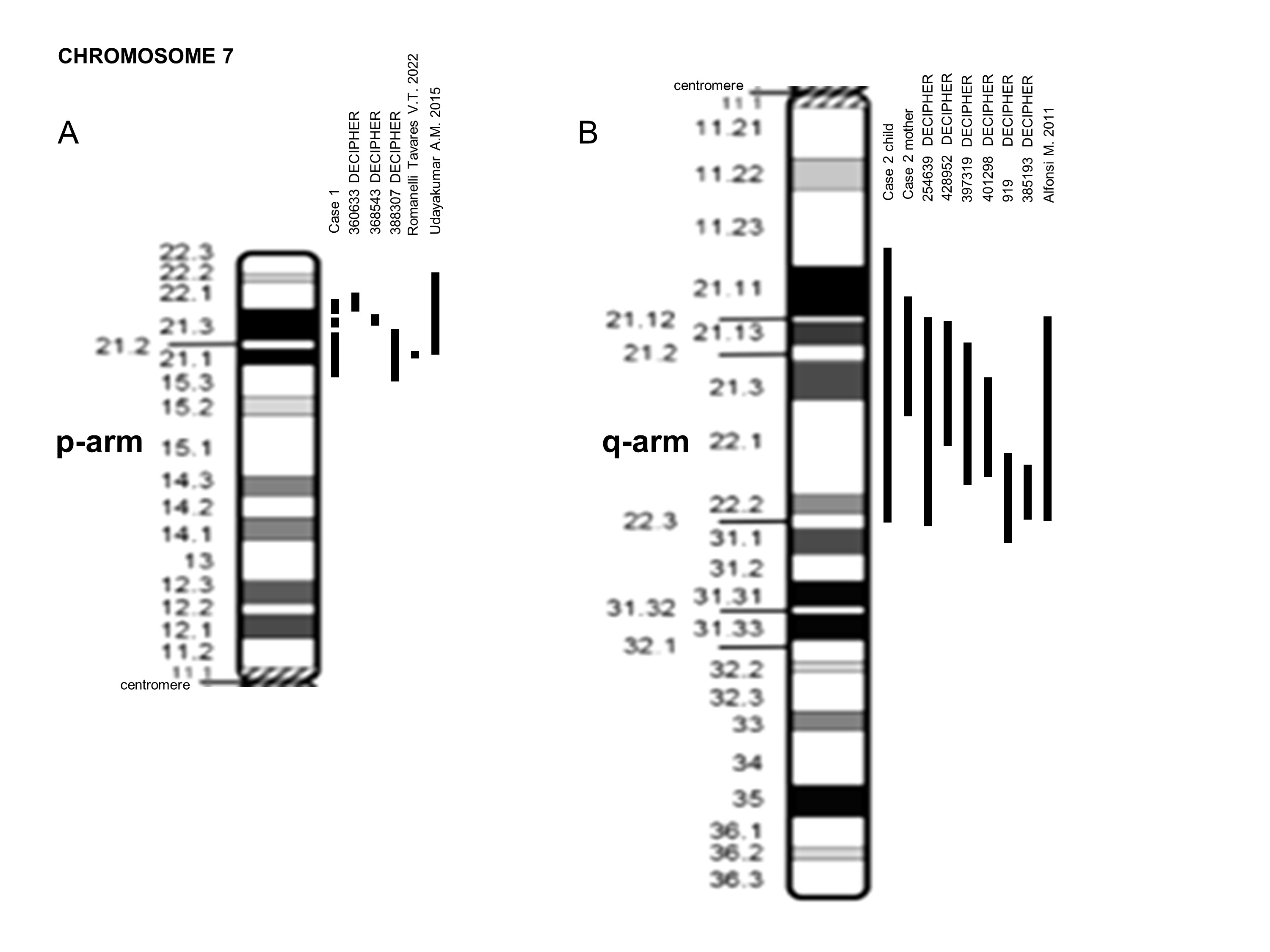

Supplement: Supplementary file 1 [file genes-14-01700-s001.zip › FigS5.jpg]
